# The autophagy-senescence axis as a threshold model of aging and therapeutic targeting

**DOI:** 10.1016/j.redox.2026.104079

**Published:** 2026-02-10

**Authors:** Md Entaz Bahar, Jin Seok Hwang, Trang Huyen Lai, Kazi-Marjahan Akter, Rizi Firman Maulidi, Deok Ryong Kim

**Affiliations:** a Department of Biochemistry and Convergence Medical Sciences and Institute of Medical Science, Gyeongsang National University, College of Medicine, Jinju, South Korea; b College of Pharmacy and Research Institute of Pharmaceutical Sciences, Gyeongsang National University, Jinju, South Korea

**Keywords:** Autophagy, Senescence, Cellular stress, Threshold-model, Targeted senotherapy

## Abstract

Autophagy and cellular senescence are fundamental stress-response programs that critically shape aging and disease progression, yet their functional relationship has remained paradoxical. Autophagy is traditionally viewed as a cytoprotective process that preserves cellular homeostasis and delays senescence. In contrast, emerging evidence demonstrates that autophagy is also indispensable for the survival and pathological activity of established senescent cells. In this review, we propose a “threshold model” to reconcile these opposing roles and to provide a unified framework linking signal transduction, organelle quality control, and therapeutic intervention. According to this model, autophagy exerts stage-dependent functions governed by stress intensity and disease progression. Below a critical damage threshold, robust autophagic flux suppresses senescence initiation by maintaining mitochondrial integrity, limiting oxidative stress, and preserving proteostasis. Once this threshold is exceeded, autophagy is functionally reprogrammed to sustain the metabolic and biosynthetic demands of senescent cells, including production of the senescence-associated secretory phenotype (SASP). We highlight key signaling nodes that regulate this transition, including mTORC1, AMPK, p53, and p62, as well as spatial and organelle-specific mechanisms such as the TOR–autophagy spatial coupling compartment (TASCC), mitophagy failure, lipophagy blockade, and aberrant nucleophagy. These processes converge on innate immune pathways, notably cGAS–STING and NF-κB signaling, to drive chronic inflammation and tissue dysfunction. Importantly, we extend this mechanistic framework to clinical translation, synthesizing evidence from ongoing trials in cancer, neurodegeneration, metabolic liver disease, and fibrosis. We argue that effective targeting of the autophagy–senescence axis requires precision gerontology, integrating dynamic biomarkers to guide stage-specific interventions—autophagy activation for prevention and autophagy inhibition or senolysis for established disease. This threshold-based perspective provides a rational foundation for next-generation therapeutic strategies targeting aging and age-related disorders.

## Highlights

• Autophagy and senescence have opposing but interconnected roles in aging and disease.
• Autophagy suppresses senescence at early stages but supports senescent cell survival later.
• A threshold model explains stress- and stage-dependent autophagy reprogramming.
• Precision targeting of autophagy enables stage-specific anti-aging and disease therapies.

## 1 Introduction

Macroautophagy and cellular senescence are evolutionarily conserved stress-response programs that play essential roles in maintaining cellular homeostasis and tissue integrity [1]. Macroautophagy, hereafter referred to as autophagy, represents the principal intracellular degradation pathway through which cytoplasmic constituents are recycled. First described by Christian de Duve [2], and mechanistically elucidated by Yoshinori Ohsumi [3], autophagy functions as a cytoplasmic quality-control system by sequestering damaged organelles and misfolded proteins into double-membrane vesicles termed autophagosomes, which subsequently fuse with lysosomes for degradation This process generates metabolic intermediates that support cell survival during nutrient deprivation and prevents the accumulation of cytotoxic cellular debris. Cellular senescence, originally described by Leonard Hayflick in the 1960s [4], is defined as a stable and irreversible arrest of the cell cycle. Triggered by telomere attrition (replicative senescence) or diverse cellular stresses (premature senescence), this program functions as a potent tumor-suppressive mechanism by permanently restricting the proliferation of cells harboring potentially oncogenic damage [5].

Historically, senescence has been studied as a terminal growth-arrest program, whereas autophagy has been viewed primarily as a mechanism for sustaining metabolic viability. However, accumulating evidence over the past decade has revealed that these processes are tightly interconnected within a complex regulatory network. Basal autophagic flux declines with aging, a change that correlates closely with the accumulation of senescent cells across multiple tissues [6]. Under physiological conditions, robust autophagy delays the onset of senescence by mitigating oxidative stress, preserving mitochondrial integrity, and limiting cellular damage that would otherwise trigger permanent cell-cycle arrest [7]. Accordingly, impaired autophagic capacity is now widely recognized as a key contributor to the aging phenotype, driving metabolic dysfunction, neurodegeneration, and organismal frailty [8].

Despite this protective role, a fundamental paradox has emerged that challenges the simplistic classification of autophagy as an exclusively anti-aging mechanism. Although autophagy suppresses senescence initiation, growing evidence indicates that it is also required for the maintenance and persistence of established senescent cells [9]. Senescent cells remain metabolically active and adopt a hypersecretory state characterized by the senescence-associated secretory phenotype (SASP), which includes pro-inflammatory cytokines, growth factors, and proteases that disrupt tissue homeostasis [10,11]. To sustain this energetically demanding phenotype, senescent cells exploit the autophagic machinery to recycle intracellular components into amino acids and metabolic substrates [12]. Consequently, autophagy exhibits a context-dependent duality: a protective mechanism in healthy cells that becomes a pro-survival pathway supporting dysfunctional, inflammation-driving senescent cells [13].

This paradox raises a critical unresolved question: how does autophagy switch from suppressing senescence to sustaining it? This ambiguity presents a major challenge for therapeutic intervention, as systemic autophagy inhibition risks neurotoxicity, whereas autophagy activation may rejuvenate healthy tissues while inadvertently promoting the survival of senescent or malignant cells. The molecular determinants governing this functional transition—specifically, the point at which autophagy shifts from a cytoprotective barrier to a pro-survival mechanism—remain poorly defined. In this review, we propose a “**threshold model”** to address this gap. We posit that the functional relationship between autophagy and senescence is dynamically regulated by the magnitude of cellular stress and the temporal stage of disease progression. We first examine the molecular switches underlying this transition, with particular emphasis on signaling crosstalk involving mTORC1, p53, and organelle-specific autophagic failures such as defective mitophagy. Finally, we discuss the translational implications of this framework, illustrating how the threshold model supports a stage-dependent therapeutic strategy—leveraging autophagy induction for disease prevention and autophagy inhibition as a senolytic approach in aging-associated disorders and cancer.

## 2 The evolving landscape of stress biology: autophagy and senescence crosstalk

Despite seminal discoveries by Christian de Duve, who coined the term *autophagy* in 1963, and Leonard Hayflick, who defined the replicative limits of somatic cells in 1961, the fields of autophagy and cellular senescence developed largely along parallel and non-intersecting trajectories for several decades [14]. Autophagy was historically conceptualized as a cytoplasmic housekeeping process responsible for nutrient recycling and intracellular quality control, whereas cellular senescence was viewed as a nuclear event marking a terminal and irreversible state of growth arrest induced by telomere erosion or genotoxic stress.

This compartmentalized view has undergone a substantial revision. Rather than functioning as isolated processes, autophagy and senescence are now recognized as interdependent components of cellular homeostasis. Although the 2016 Nobel Prize awarded to Yoshinori Ohsumi underscored the fundamental importance of autophagic machinery, a critical question remains unresolved: how does the same recycling system that preserves cellular fitness influence the persistence and fate of senescent cells?

Cellular viability relies on a finely regulated balance between biosynthesis and degradation which is commonly referred to as proteostatic homeostasis, which progressively deteriorates with aging. Within this context, autophagy and senescence operate as complementary but temporally distinct protective mechanisms. Under physiological conditions, robust autophagic flux delays the onset of senescence by limiting oxidative stress, preserving mitochondrial integrity, and preventing the accumulation of cellular damage that would otherwise trigger permanent cell-cycle arrest [8,15]. When damage becomes irreparable, senescence is engaged as a failsafe program that enforces durable proliferative arrest, thereby safeguarding tissue integrity.

Recent evidence, however, reveals that the relationship between autophagy and senescence is not linear but biphasic. While autophagy suppresses senescence initiation in early stages, it is subsequently co-opted to support the metabolic and secretory demands of fully established senescent cells. To reconcile this apparent contradiction, we propose a threshold model of regulation. In this framework, the functional output of autophagy is dynamically determined by the magnitude of cellular stress and the temporal stage of pathological progression. Below a critical damage threshold, autophagy acts as a cytoprotective barrier against aging; beyond this threshold, it is repurposed as a pro-survival mechanism that sustains senescent cell viability.

### 2.1 Historical convergence: from parallel tracks to a unified network

**Fig. 1:**
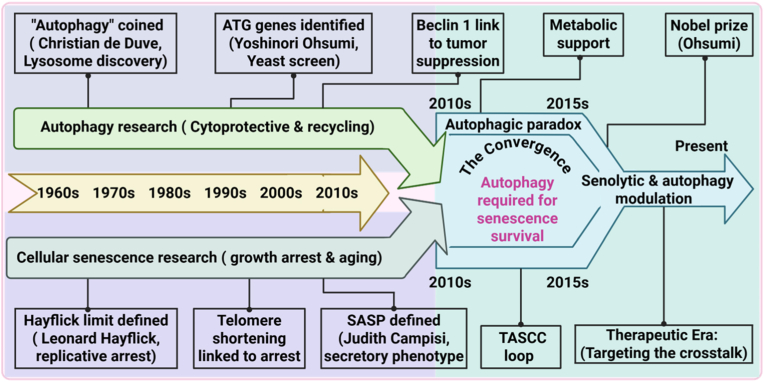
Historical convergence of autophagy and senescence research (1960–**;Present).** This timeline depicts the parallel evolution of autophagy (upper track) and cellular senescence (lower track) as largely independent research fields from the 1960s onward. Convergence occurs in the late 2000s with the identification of SASP and the recognition that senescent cells depend on autophagy for metabolic support. These discoveries established a unified mechanistic framework linking lysosomal recycling to senescence biology and provided the conceptual basis for contemporary senolytic and autophagy-targeted therapeutic strategies.

To contextualize the proposed threshold model, it is essential to trace the historical convergence of autophagy and senescence research, which has transformed two once-independent fields into a unified signaling network governing cellular fate decisions (Fig. 1).

#### 2.1.1 Period of definition (1960s)

The conceptual foundations of senescence and autophagy were established through two independent landmark discoveries. In 1961, Leonard Hayflick overturned the prevailing assumption of cellular immortality by defining the finite replicative capacity of somatic cells, now termed the *Hayflick limit*, thereby establishing senescence as a programmed cellular outcome [4]. Shortly thereafter, in 1963, Christian de Duve characterized the lysosome and introduced autophagy as a regulated process of intracellular self-digestion, providing the first mechanistic framework for lysosome-mediated recycling [2].

#### 2.1.2 Period of specialization (1990s)

For several decades, these fields evolved largely in isolation. Autophagy research focused predominantly on nutrient stress responses in yeast, culminating in Yoshinori Ohsumi's identification of the autophagy-related (ATG) genes in 1993. In parallel, senescence research centered on tumor suppression, linking irreversible growth arrest to telomere erosion and activation of cyclin-dependent kinase inhibitors, particularly the p16^INK4a^ pathway [16]. During this phase, autophagy was viewed as a metabolic survival mechanism, whereas senescence was regarded as a static, nuclear-driven endpoint.

#### 2.1.3 Era of convergence (2000s–Present)

The conceptual separation between autophagy and senescence began to dissolve in the late 2000s. The identification of the SASP by Campisi and colleagues in 2008 demonstrated that senescent cells are metabolically active and exert profound paracrine effects on tissue microenvironments [11,17]. Subsequently, seminal studies by White, Narita, and others (2009–2011) revealed that senescent cells depend on autophagy to sustain SASP production, uncovering the so-called *autophagic paradox*—the coupling of lysosomal degradation to chronic inflammation and age-related pathology [9,18,19].

### 2.2 Pre-threshold phase: autophagy as a protective barrier

#### 2.2.1 Mechanism

**Fig. 2:**
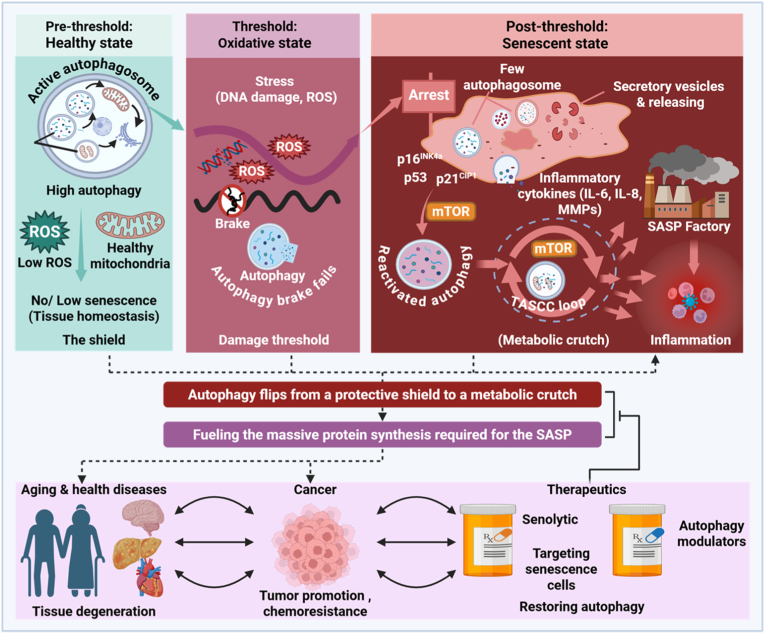
The autophagy-senescence threshold model. Cellular mechanisms (upp**er panel).***Pre-threshold (left):* In homeostatic cells, elevated autophagic flux maintains mitochondrial quality and limits reactive oxygen species (ROS) accumulation, thereby preserving cellular function and preventing damage-induced senescence. *Threshold (center):* When cumulative stress surpasses the degradative capacity of autophagy, this protective barrier collapses, triggering irreversible cell-cycle arrest through activation of the p53–p21 and p16 pathways. *Post-threshold (right):* Following the establishment of senescence, autophagy is reactivated but functionally repurposed to support cellular survival by recycling intracellular components to meet the heightened metabolic and biosynthetic demands of the senescence-associated secretory phenotype (SASP). **Clinical implications (lower panel).** Failure of the autophagic quality-control barrier promotes tissue degeneration and functional decline, whereas autophagy repurposing in senescent cells sustains chronic inflammation and facilitates tumor progression. This model underscores the need for stage-specific therapeutic strategies: autophagy activation to reinforce cytoprotective barriers in disease prevention, and autophagy inhibition or senolytic interventions to selectively eliminate senescent cells in established pathology.

During homeostatic conditions, basal autophagy constitutes a primary defense against cellular aging. Acting as a high-fidelity quality-control system, autophagy selectively removes protein aggregates, lipofuscin deposits, and dysfunctional organelles. In particular, mitophagy eliminates depolarized mitochondria before excessive production of reactive oxygen species (ROS), thereby preserving mitochondrial integrity and limiting oxidative stress (Fig. 2).

#### 2.2.2 Outcome

By maintaining low intracellular ROS levels and preserving organelle function, autophagy raises the threshold required to activate the DNA damage response (DDR). Enhanced autophagic flux during this phase effectively delays senescence onset and sustains cellular fitness [6].

### 2.3 Threshold phase: excessive stress and functional switching

Cellular transformation occurs when cumulative damage induced by genotoxic stress, irradiation, or chronic inflammation overwhelms lysosomal clearance capacity. This critical inflection point defines the threshold phase (Fig. 2)**.**

#### 2.3.1 Mechanism

Once damage exceeds the cell's degradative capacity, a transient suppression of autophagic activity is frequently observed. This temporary interruption permits the stabilization of p53 and the accumulation of metabolic stress signals, facilitating activation of p21 and p16^INK4a^. These events collectively enforce irreversible cell-cycle arrest and commitment to the senescent state [20].

#### 2.3.2 Outcome

At this stage, the cytoprotective barrier provided by autophagy collapses, and the cell transitions from a potentially repairable stress state to an established senescent phenotype.

### 2.4 Post-threshold phase: autophagy as a pro-survival metabolic program

Once senescence is fully established, autophagy is reactivated; however, its functional role is fundamentally reprogrammed (Fig. 2).

#### 2.4.1 Mechanism

Senescent cells exhibit an exceptionally high biosynthetic burden due to sustained SASP production, characterized by elevated secretion of IL-6, IL-8, and matrix metalloproteinases. To meet these metabolic demands in the absence of proliferation, senescent cells enter a hypermetabolic state and repurpose autophagic machinery. This process involves spatial coupling of autophagy with mTORC1 signaling through the TOR–autophagy spatial coupling compartment (TASCC), enabling efficient conversion of intracellular proteins into free amino acids that fuel SASP synthesis (see Section 3).

#### 2.4.2 Outcome

In the post-threshold phase, autophagy no longer functions as a protective mechanism but instead supports the survival and pathogenic activity of senescent cells. By sustaining chronic inflammatory signaling, this repurposed autophagy accelerates tissue degeneration and promotes tumor progression [12].

## 3 The molecular machinery: from signaling switches to organelle failure

**Fig. 3:**
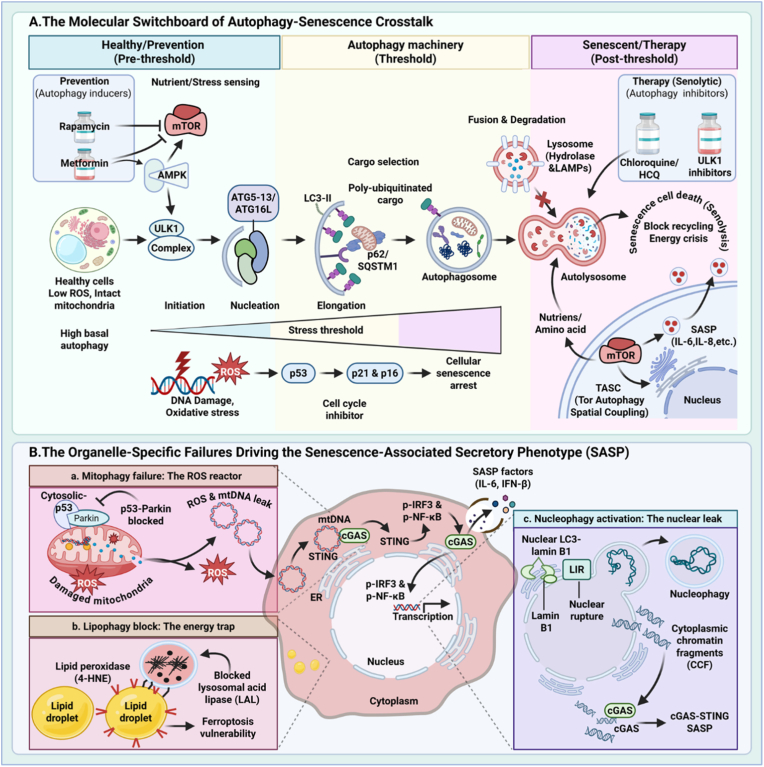
The intracellular decision nodes that regulate the transition from cytoprotective autophagy (pre-thresh**old state) to autophagy-dependent maintenance of senescence (post-threshold state)**. **A. Signaling circuitry of autophagy–senescence crosstalk.** This panel illustrates the biphasic, stage-dependent function of autophagy during senescence progression. *Pre-threshold (left):* In homeostatic cells, elevated autophagic flux preserves mitochondrial integrity and limits reactive oxygen species (ROS) accumulation, thereby preventing activation of cell-cycle arrest programs. *Threshold (center):* When cumulative metabolic stress—including DNA damage and oxidative burden—exceeds the degradative capacity of autophagy, the damage threshold is breached, leading to irreversible cell-cycle arrest mediated by p53–p21 and p16^INK4a^ signaling. *Post-threshold (right):* In established senescent cells, autophagy is reactivated and spatially coupled to mTORC1 signaling. This reprogrammed autophagy recycles intracellular components to sustain the high biosynthetic demands of the senescence-associated secretory phenotype (SASP), thereby promoting chronic tissue inflammation. **B. Organelle-specific autophagy defects driving SASP induction.** This panel depicts three major failures in selective autophagy that convert metabolic dysfunction into inflammatory signaling within senescent cells. *(a) Mitophagy failure:* In healthy cells, the PINK1–Parkin pathway mediates clearance of damaged mitochondria. In senescence, cytoplasmic p53 impedes Parkin recruitment, resulting in accumulation of enlarged, dysfunctional mitochondria. These organelles release oxidized mitochondrial DNA (mtDNA) into the cytosol, activating the cGAS–STING pathway and driving NF-κB–dependent cytokine production, including IL-6. *(b) Lipophagy blockade:* Downregulation of lipid droplet–associated receptors (e.g., PLIN2) prevents lipid mobilization, leading to accumulation of lipid peroxides and lipofuscin. This lysosomal congestion promotes metabolic inflexibility and increases susceptibility to ferroptosis. *(c) Nucleophagy hyperactivation:* Autophagy protein LC3 aberrantly targets Lamin B1, resulting in partial degradation of the nuclear envelope and generation of cytoplasmic chromatin fragments (CCFs). These CCFs activate the cGAS–STING pathway independently, reinforcing interferon signaling and sustaining the SASP-driven inflammatory loop.

The preceding section defined the threshold model as a temporal framework governing the functional transition of autophagy during aging. Here, we dissect the molecular pathways that actively drive the shift from cytoprotective autophagy to senescence maintenance. As summarized in Fig. 3, this transition is controlled at two interrelated levels. First, a network of intracellular signaling nodes, including mTORC1, p53, and the autophagy adaptor p62, integrates metabolic and stress signals to determine cellular fate (Fig. 3A). Second, defects in selective autophagy downstream of these signaling events translate metabolic imbalance into a sustained inflammatory output, manifested as SASP (Fig. 3B).

### 3.1 The kinetic switch: biphasic autophagy dynamics

The shift from homeostatic condition to cellular senescence is non-linear and entails a specific kinetic “switch” in autophagic flow, influenced by the duration of stress and subcellular spatial reorganization. During the first induction phase, such as after acute DNA damage, autophagy is frequently temporarily inhibited. This inhibition is mechanistically mediated by cytoplasmic p53, which directly obstructs the ULK1–FIP200 complex to prevent autophagosome initiation [21]. This brief cessation has two functions: it enables the accumulation of DDR intermediates necessary for implementing irreversible cell-cycle arrest, and it allows for the significant cytoskeletal remodeling and cellular hypertrophy typical of senescent morphology [22].

This suppression is ephemeral. Upon the establishment of the senescent phenotype, the elevated metabolic requirements of the SASP requires a vigorous reactivation of autophagy. The activation is prompted by the establishment of the TASCC, wherein mTORC1 and autolysosomes are spatially associated to facilitate nutrient recycling especially for SASP synthesis [23]. Thus, the threshold signifies a functional inversion: autophagy transitions from a universal degradative quality-control mechanism to a specialized biosynthetic support system that maintains the survivability and inflammatory output of senescent cells [24].

### 3.2 Intracellular signaling nodes: the decision circuity

The conversion of a damage-responsive cell into a senescent, inflammation-producing cell is not the result of passive degeneration but reflects an actively regulated rewiring of intracellular signaling pathways.

#### 3.2.1 The TASCC loop: uncoupling growth control from energy stress

One of the defining molecular alterations in senescence is the breakdown of the canonical coupling between energy availability and cellular growth. Under physiological conditions, the energy sensor AMPK and the anabolic regulator mTORC1 function as mutually exclusive signaling switches. Energetic stress activates AMPK, leading to phosphorylation of the TSC1/2 complex and subsequent inhibition of mTORC1, thereby suppressing biosynthesis and conserving cellular resources [12,25].

In senescent cells, this regulatory logic is subverted through the formation of the TOR–autophagy spatial coupling compartment (TASCC). As first described by Narita et al. (2011), autolysosomes and active mTORC1 are spatially reorganized into a distinct perinuclear compartment adjacent to the trans-Golgi network. This architecture sustains localized mTORC1 signaling despite global indicators of cellular stress.

##### 3.2.1.1 Mechanism

The TASCC operates as a self-contained metabolic recycling unit. Autolysosomal degradation of intracellular components generates free amino acids, particularly arginine and leucine. Rather than diffusing into the cytosol, these amino acids are rapidly sensed by Rag GTPases at the lysosomal membrane, triggering immediate reactivation of mTORC1 within the same compartment [[26], [27], [28]].

##### 3.2.1.2 Outcome

This spatially restricted coupling enables senescent cells to maintain high rates of protein synthesis independent of extracellular nutrient availability. As a result, senescent cells can sustain continuous production of SASP components, including IL-6 and IL-8, by recycling endogenous cellular material.

#### 3.2.2 The biphasic gatekeeper: p53

The tumor suppressor p53 functions as a central regulator of the autophagy–senescence threshold, exerting opposing effects on autophagy depending on its subcellular localization and activation state [26,29].

##### 3.2.2.1 Nuclear p53 (the shield)

Under conditions of low to moderate stress, p53 is predominantly localized to the nucleus, where it transcriptionally activates pro-autophagic and stress-adaptive genes, including *DRAM1* and *sestrin1/2*. Through these targets, nuclear p53 promotes autophagic clearance of damaged organelles and macromolecules, thereby preserving cellular integrity and reinforcing the pre-threshold, cytoprotective state.

##### 3.2.2.2 Cytoplasmic p53: enforcement of irreversible arrest

Severe genotoxic stress induces relocalization of p53 from the nucleus to the cytoplasm, where it exerts a non-transcriptional inhibitory effect on autophagy. Cytoplasmic p53 directly interacts with FIP200, a core component of the ULK1 initiation complex, thereby blocking autophagosome formation at its earliest stage [30]. This transient but decisive suppression of autophagy facilitates stabilization of damage signals and is required for the establishment of irreversible cell-cycle arrest. Consequently, cytoplasmic p53 functions as a molecular brake that enforces commitment to the senescent state.

#### 3.2.3 The p62/SQSTM1 hub: from cargo receptor to signaling scaffold

p62 (SQSTM1) is classically defined as an autophagy adaptor that delivers ubiquitinated proteins to autophagosomes for degradation. Under conditions in which autophagic flux is saturated or impaired, p62 escapes degradation and progressively accumulates, undergoing oligomerization into cytoplasmic aggregates.

##### 3.2.3.1 Mechanism

Accumulated p62 transitions from a cargo shuttle to a signaling scaffold. p62 oligomers recruit the E3 ubiquitin ligase TRAF6, promoting K63-linked polyubiquitination of the IKK complex and subsequent activation of downstream inflammatory signaling.

##### 3.2.3.2 Outcome

This signaling cascade culminates in nuclear translocation of NF-κB, a master transcriptional regulator of inflammatory and SASP-related genes [28]. Through this mechanism, p62 provides a direct molecular link between impaired autophagic clearance and sustained pro-inflammatory signaling.

#### 3.2.4 Secretory autophagy: the non-canonical delivery system

Secretory autophagy utilizes the fundamental molecular apparatus to transport cytosolic proteins lacking N-terminal signal peptides, facilitating unusual protein secretion. This differs from canonical autophagy, which culminates in lysosomal degradation.

##### 3.2.4.1 Mechanism

The LC3 conjugation system and the ATG5–ATG12–ATG16L1 complex function not as markers for degradation, but as signals for encapsulation. These proteins transport certain SASP components, such as IL-1β, HMGB1, and ferritin, into double-membrane vesicles called “secretory autophagosomes” [31,32]. These vesicles are not connected to lysosomes; instead, they move toward the cell periphery. They attach to the SNARE protein SEC22B to directly fuse with the plasma membrane or combine with multivesicular bodies (MVBs). This releases their payload, which is stored in extracellular vesicles (EVs) or exosomes [33]. This mechanism turns the autophagic machinery from a mechanism for getting rid of trash into a way to transfer vesicles. This lets the SASP release a lot of material.

##### 3.2.4.2 Outcome

Upon activation of secretory autophagy, the autophagic machinery transitions from a cell-autonomous protective mechanism to a contributor to tissue dysfunction. This pathway facilitates paracrine senescence by actively translocating elevated levels of inflammatory cytokines and damage-associated molecular patterns (DAMPs) into the microenvironment. Secretory autophagy releases extracellular vesicles, which are then absorbed by adjacent healthy cells. These vesicles then induce DNA damage responses and secondary senescence, commonly referred to as the bystander effect [34]. This continuous release induces chronic inflammation and establishes a chemotactic gradient that attracts immune cells. Consequently, post-threshold autophagy not only sustains the viability of senescent cells but also actively contributes to systemic tissue degeneration [35].

#### 3.2.5 The proteostatic collapse: UPS-autophagy crosstalk

It is essential to recognize that autophagy operates in conjunction with the ubiquitin-proteasome system (UPS). The UPS manages transient proteins, whereas autophagy eliminates large clumps. During senescence, this alliance disintegrates.

##### 3.2.5.1 Mechanism: compensatory failure

Senescence is marked by a gradual reduction in proteasomal catalytic activity, leading to the buildup of oxidized and polyubiquitinated proteins. In optimal settings, autophagy functions as a compensatory mechanism (via aggrephagy) when the proteasome is overloaded [36]. Nonetheless, in the post-threshold condition, this compensation is ineffective. The extensive quantity of proteasome-resistant substrates overwhelms the autophagic system, resulting in the buildup of p62 oligomers that remain uncleared. This illustrates a “traffic jam” in which the malfunction of the primary disposal system (UPS) obstructs the secondary system (autophagy).

##### 3.2.5.2 Outcome: toxicity and synthetic lethality

This aggregation possesses twofold toxicity: it imposes a mechanical load on the lysosomal system and actively enhances NF-kB–dependent inflammatory signaling, hence exacerbating the SASP [37]. Thus, the concurrent disruption of proteasomal degradation and the redirection of autophagy towards SASP support results in a catastrophic breakdown of proteostasis. This synchronized failure elucidates why senescent cells exhibit heightened sensitivity to autophagy inhibitors; having forfeited their principal degradation pathway (the proteasome), they rely exclusively on the “life-support” provided by the repurposed autophagy system. Inhibiting this ultimate pathway induces rapid apoptosis, a phenomenon referred termed as synthetic lethality [38].

### 3.3 Organelle-specific dysfunctions: initiating signals for the SASP

In aging cells, the decline of bulk macroautophagy is compounded by selective failures in organelle-specific autophagy. These defects do not merely result in passive accumulation of damaged structures; rather, dysfunctional organelles actively serve as sources of inflammatory signaling that drive senescence-associated phenotypes.

#### 3.3.1 Mitophagy failure: amplification of oxidative and innate immune signaling

Mitochondria represent a major source of age-associated oxidative stress. In healthy cells, depolarized mitochondria are selectively removed via the PINK1–Parkin mitophagy pathway. This quality-control mechanism deteriorates with age, in part due to cytoplasmic p53–mediated inhibition of Parkin translocation to damaged mitochondria [39].

**Outcome:** Defective mitophagy results in accumulation of enlarged, dysfunctional mitochondria that exhibit impaired respiration but generate excessive ROS.

**cGAS-STING activation:** Structural compromise of these mitochondria permits the release of oxidized mitochondrial DNA (mtDNA) into the cytosol. Cytosolic mtDNA is sensed by cGAS, triggering activation of the STING pathway and inducing sustained production of interferon-β and IL-6, thereby reinforcing chronic inflammatory signaling associated with aging and senescence [40].

#### 3.3.2 Lipophagy blockade: metabolic inflexibility and lipid toxicity

Aging cells frequently accumulate lipid droplets (LDs), adopting a foamy morphology. This phenotype arises from impaired lipophagy, driven by downregulation of LD-associated receptors such as perilipin 2 (PLIN2) or dysfunction of lysosomal acid lipase (LAL).

**Outcome:** Lipophagy blockade traps cells in a state of metabolic inflexibility: despite increased energetic demand imposed by the SASP, stored lipids cannot be mobilized. Moreover, accumulated lipids undergo ROS-mediated peroxidation, contributing to the formation of lipofuscin—an indigestible protein–lipid aggregate that impairs lysosomal function, further suppresses autophagic flux, and restricts cell motility [41].

**Ferroptosis susceptibility:** Progressive accumulation of lipid peroxides increases sensitivity of senescent cells to ferroptosis, an iron-dependent form of regulated cell death. This vulnerability has emerged as a promising therapeutic target for senolytic strategies, particularly through inhibition of glutathione peroxidase 4 (GPX4) [42].

#### **3.3.3** **Nucleophagy hyper-activation:** nuclear integrity loss and DNA sensing

In contrast to blocked mitophagy and lipophagy, nucleophagy is aberrantly activated during senescence. A hallmark of this process is the progressive loss of Lamin B1 from the nuclear lamina. Autophagy protein LC3 directly associates with Lamin B1–containing nuclear fragments, targeting them for lysosomal degradation [43].

**Outcome:** Excessive nucleophagy compromises nuclear envelope integrity, allowing chromatin fragments to leak into the cytoplasm. These cytoplasmic chromatin fragments (CCFs) function as potent activators of innate immune signaling.

**Feed-forward inflammatory loop:** Similar to mtDNA, CCFs activate the cGAS–STING pathway independently, amplifying SASP gene expression. This establishes a self-reinforcing loop in which nuclear degradation promotes cytosolic DNA sensing, STING activation, and escalating inflammatory output [44].

#### 3.3.4 Chaperone-mediated autophagy failure lowers the senescence threshold

Chaperone-mediated autophagy (CMA) is an important but underappreciated mechanism for maintaining cellular health as individuals age, along with macroautophagy [45]. Chaperone-mediated autophagy (CMA) selectively targets and degrades cytosolic proteins containing KFERQ-like domains, including critical regulators of stress response and metabolism such as p53, GAPDH, and hexokinase 2 [46]. This is not the same as bulk autophagy. As people get older, lysosomal LAMP2A levels decrease, which reduces CMA efficiency [47]. This causes oxidized and defective proteins to build up, which bulk autophagy alone cannot clear [48].

The inability of CMA to lower the senescence threshold exacerbates proteotoxic stress and metabolic imbalance, thereby facilitating early entry into irreversible growth arrest. Once senescence sets in, CMA activity remains low, indicating that the cell relies on macroautophagy to survive. These findings indicate that CMA dysfunction serves as an initial, upstream factor in “Threshold Crossing” and underscore the necessity of incorporating several autophagy pathways in the formulation of therapeutics aimed at senescence.

#### 3.3.5 Redox compartmentalization: the aggregate threshold

Mitochondrial dysfunction is often considered the primary trigger for the senescence threshold; nevertheless, redox stress is inherently localized inside certain organelle microenvironments. The endoplasmic reticulum (ER) significantly contributes to this overall burden, particularly via the ERO1α-PDI pathway during protein folding. This pathway produces ROS as a byproduct and initiates the unfolded protein response (UPR), which halts the cell cycle [49]. The age-related reduction in pexophagy, the mechanism responsible for the degradation and elimination of peroxisomes, results in the accumulation of dysfunctional organelles that release hydrogen peroxide (H_2_O_2_) and disrupt the redox equilibrium in the cytosol [50]. Thus, the senescence “threshold” is not defined by a singular global signal; rather, it is influenced by the cumulative impacts of mitochondrial superoxide, ER oxidative stress, and peroxisomal leakage, which collectively exceed the cellular antioxidant defenses [51].

## 4 Context-dependent outcomes: the dual role of the autophagy–senescence axis in disease

The therapeutic consequences of the autophagy–senescence axis are highly context dependent. The same molecular pathways that preserve tissue integrity under physiological conditions may, in specific disease settings, promote pathology. This dichotomy explains why pharmacological activation of autophagy can extend lifespan and health span in otherwise healthy organisms, yet exacerbate disease progression and mortality in advanced malignancies [52]. Understanding this duality is essential for designing stage- and tissue-specific therapeutic strategies.

### 4.1 Cancer: a driver of dormancy and relapse

**Fig. 4:**
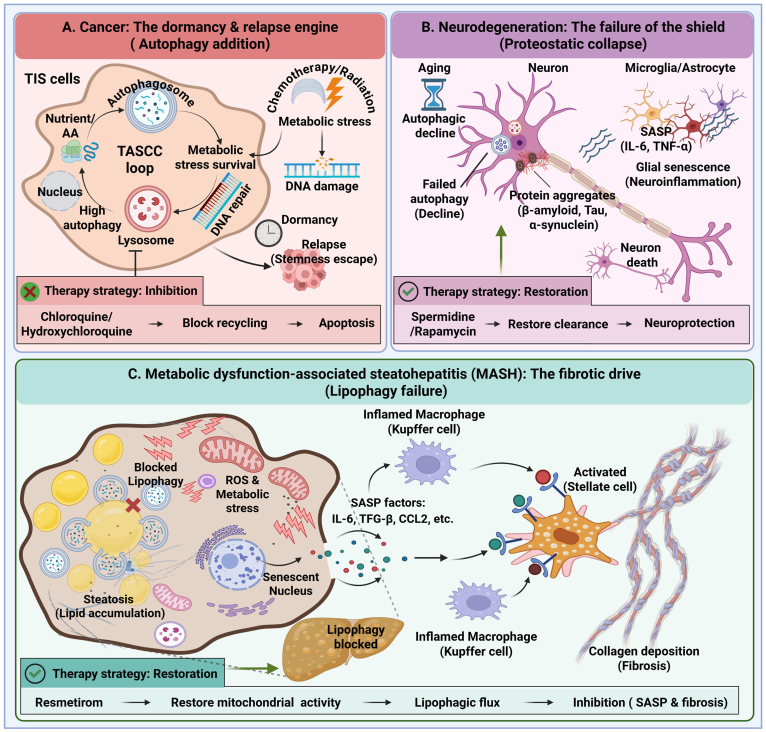
Context-dependent outcomes of the autophagy–senesc**ence axis.** This diagram illustrates why clinical modulation of autophagy must be tailored to disease context and stage. **(A) Cancer: autophagy-driven dormancy and relapse.** In malignant cells, autophagy is frequently hyperactivated to sustain survival under therapeutic stress. Cytotoxic treatments induce therapy-induced senescence (TIS), during which tumor cells exploit the TASCC loop to recycle intracellular nutrients and facilitate DNA repair. This autophagy dependence enables entry into a metabolically dormant state that permits treatment evasion, followed by senescence escape and aggressive relapse. *Therapeutic strategy:* pharmacological inhibition of autophagy (e.g., hydroxychloroquine) to disrupt nutrient recycling and selectively eliminate dormant tumor cells. **(B) Neurodegeneration: collapse of the cytoprotective barrier.** In post-mitotic neurons, age-associated decline in autophagic flux leads to accumulation of aggregation-prone proteins, including amyloid-β, tau, and α-synuclein. Resultant proteotoxic stress induces secondary senescence in glial cells (microglia and astrocytes), driving neuroinflammation and progressive neuronal loss. *Therapeutic strategy:* restoration of autophagic clearance (e.g., spermidine or rapamycin) to remove toxic aggregates and re-establish cellular homeostasis. **(C) Metabolic disease/MASH: lipophagy failure and fibrogenesis.** In metabolic dysfunction–associated steatohepatitis (MASH), impaired hepatocyte lipophagy prevents lysosomal degradation of lipid droplets, leading to lipotoxic stress and senescence induction. Senescent hepatocytes secrete profibrotic SASP factors, such as TGF-β, which activate quiescent hepatic stellate cells into collagen-producing myofibroblasts. *Therapeutic strategy:* autophagy restoration via THR-β activation (e.g., resmetirom) to enhance mitochondrial function and lipophagic flux, thereby clearing toxic lipids and preventing senescence-driven fibrosis.

Therapy-induced senescence (TIS) is a frequent but clinically problematic outcome of chemotherapy and radiotherapy. Although senescence initially suppresses tumor growth, senescent cancer cells can persist as a latent reservoir with long-term pathological consequences. Unlike normal cells, which often undergo apoptosis in response to genotoxic stress, malignant cells—particularly those harboring oncogenic RAS mutations—exhibit a strong dependence on autophagy to survive therapy-induced metabolic stress [53]. These senescent cancer cells maintain elevated catabolic activity through autophagy-driven nutrient recycling, supported by the TASCC loop described in Fig. 4A. This metabolic adaptation enables DNA repair and sustains viability under cytotoxic conditions [12,54].

As a result, autophagy supports a prolonged state of tumor dormancy. Importantly, accumulating evidence indicates that autophagy is required to maintain stem-like properties in senescent cancer cells, facilitating escape from growth arrest and re-entry into the cell cycle, ultimately leading to aggressive, therapy-resistant relapse [55].

Therapeutically, disrupting this dependency represents a rational intervention. Lysosomotropic agents such as hydroxychloroquine (HCQ; e.g., **NCT06949982)** inhibit autophagic flux, depriving senescent tumor cells of recycled nutrients. This forces cells out of a protected senescent state and promotes senolytic elimination via apoptosis.

### 4.2 Neurodegeneration: failure of the protective barrier

In contrast to cancer, age-related neurodegenerative disorders—including Alzheimer's and Parkinson's diseases—arise primarily from collapse of the pre-threshold cytoprotective barrier.

#### 4.2.1 Mechanism

Neurons are post-mitotic, energetically demanding cells that rely on constitutive autophagy to maintain proteostasis by clearing aggregation-prone proteins such as amyloid-β, tau, and α-synuclein. With aging, basal autophagic capacity progressively declines, leading to impaired protein turnover and accumulation of neurotoxic aggregates in Fig. 4B [56].

#### 4.2.2 Outcome

Pathology rapidly extends beyond neurons to glial populations. Accumulated protein aggregates induce functional reprogramming of microglia and astrocytes toward a senescence-like, pro-inflammatory state. These aged glial cells secrete SASP-associated factors, including IL-6 and TNF-α, thereby amplifying neuroinflammation and accelerating neuronal loss [57]. Accordingly, therapeutic strategies in neurodegeneration aim to restore autophagic clearance. Agents such as spermidine or rapamycin (e.g., **NCT03094546**) enhance autophagy to remove toxic aggregates before irreversible inflammatory cascades are established.

### 4.3 Fibrosis: autophagy-driven scarring

The autophagy–senescence axis also plays a central role in fibrotic diseases, including metabolic dysfunction–associated steatohepatitis (MASH) and idiopathic pulmonary fibrosis (IPF). In these conditions, senescent myofibroblasts represent a major source of excessive extracellular matrix deposition. These cells exist in a hypermetabolic state, requiring large quantities of amino acids—particularly proline and glycine—for collagen biosynthesis.

Autophagy supplies this metabolic demand by degrading intracellular proteins to generate amino acid substrates that fuel extracellular matrix production [58]. Consequently, autophagy actively sustains fibrotic progression. Inhibition of autophagy in senescent myofibroblasts results in metabolic collapse and cell death, highlighting a therapeutic vulnerability. Similar to cancer, effective antifibrotic strategies are therefore focused on targeted autophagy inhibition or senolytic approaches to selectively eliminate senescent fibroblasts that constitute the fibrotic scar. These strategies are currently under evaluation in emerging preclinical models.

### 4.4 MASH: the lipophagy-senescence axis

Among age- and metabolism-associated diseases, metabolic dysfunction–associated steatohepatitis (MASH) represents one of the most clinically compelling examples of the autophagy–senescence threshold model. Progression from benign hepatic steatosis to inflammatory steatohepatitis and fibrosis is driven in large part by a selective failure of lipophagy, the autophagic degradation of lipid droplets within hepatocytes in Fig. 4C [59].

#### 4.4.1 Pre-threshold phase: lipotoxic stress accumulation

During early disease stages, excessive delivery of free fatty acids to hepatocytes leads to lipid droplet expansion. Under physiological conditions, surplus lipids are safely sequestered and eliminated through lipophagy, a process mediated in part by lipid droplet–associated receptors such as perilipin 2 (PLIN2). In MASH, however, this protective mechanism becomes overwhelmed. Impaired lysosomal acid lipase (LAL) activity prevents efficient lipid droplet degradation, resulting in progressive lipid retention and lipotoxic stress. Accumulated lipids undergo oxidative peroxidation, generating reactive aldehydes such as 4-hydroxynonenal (4-HNE), which damage mitochondrial membranes, amplify oxidative stress, and drive hepatocytes beyond the senescence threshold [60].

#### 4.4.2 Post-threshold phase: senescence-driven fibrogenesis

Once hepatocytes enter a senescent state, profound changes occur within the hepatic microenvironment. Senescent hepatocytes adopt a robust SASP characterized by elevated secretion of profibrotic mediators, including transforming growth factor-β (TGF-β) and Hedgehog ligands [61]. These factors act in a paracrine manner to activate quiescent hepatic stellate cells (HSCs), inducing their transdifferentiation into collagen-producing myofibroblasts [60]. At this stage, the context-dependent duality of autophagy becomes evident. In hepatocytes, impaired lipophagy initiates cellular damage and senescence, whereas in activated stellate cells, autophagy is upregulated to meet the energetic and biosynthetic demands of extracellular matrix production. Thus, autophagy simultaneously contributes to injury initiation and fibrosis propagation, underscoring the need for cell type– and stage-specific therapeutic modulation [58,62].

#### 4.4.3 Clinical validation: the resmetirom paradigm

The MAESTRO-NASH trial (**NCT03900429**) provided clinical validation that restoring autophagic flux represents an effective therapeutic strategy in metabolic liver disease. This study demonstrated that Resmetirom, a selective thyroid hormone receptor-β (THR-β) agonist, significantly improves steatohepatitis and attenuates fibrosis progression by reactivating hepatocyte lipophagy. FDA approval of Resmetirom (Rezfilra) in 2024 established the first pharmacological proof of concept for targeting metabolic autophagy defects in MASH. Mechanistically, THR-β activation enhances mitochondrial function and stimulates autophagy and mitophagy pathways in hepatocytes, thereby reducing lipotoxic stress and preventing transition into a post-threshold senescent state [63]. By restoring lipid clearance and metabolic homeostasis, Resmetirom interrupts the cascade linking metabolic overload to cellular aging and fibrogenesis, providing a clinically actionable demonstration of autophagy restoration as a disease-modifying intervention [64].

## 5 Clinical translation: therapeutic interventions and current trials

**Fig. 5:**
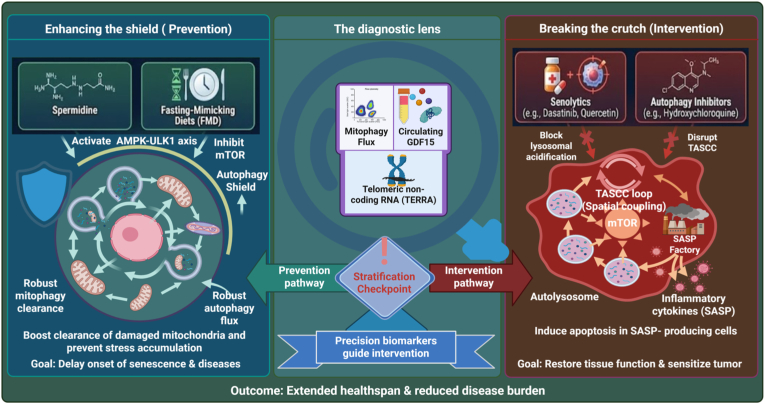
The stage-dependent therapeutic framework grounded in the mechanistic landscape of the autophagy–senesc**ence axis.***Left panel—Enhancing the shield (prevention):* In the physiological pre-threshold state, cellular homeostasis is maintained by robust autophagic flux. Preventive interventions, including spermidine and fasting-mimicking diets (FMD), activate the AMPK–ULK1 pathway while suppressing mTORC1 activity, thereby promoting mitophagy and efficient clearance of damaged organelles to limit stress accumulation. *Right panel—Breaking the crutch (intervention):* In the pathological post-threshold state, senescent cells reprogram autophagy through formation of the TOR–autophagy spatial coupling compartment (TASCC), which sustains mTORC1 signaling and fuels production of the senescence-associated secretory phenotype (SASP). Therapeutic strategies in this context shift toward senolytic agents (e.g., dasatinib and quercetin) or autophagy inhibitors (e.g., hydroxychloroquine) to impair lysosomal acidification, disrupt TASCC function, and induce apoptosis of SASP-producing cells. *Center—Diagnostic stratification:* Clinical decision-making is guided by precision biomarkers that define an individual's position along the senescence trajectory. Quantitative measures of mitophagy flux, circulating growth differentiation factor 15 (GDF15), and telomeric repeat-containing RNA (TERRA) provide critical stratification checkpoints, enabling stage-appropriate intervention and minimizing off-target risk.

Translation of the threshold model from experimental biology to clinical practice is now actively underway. A systematic survey of the ClinicalTrials.gov database identified a focused cohort of 13 registered studies directly interrogating autophagy modulation in aging- and senescence-related contexts (Table 1). The majority of these studies are interventional, reflecting a shift toward evaluating actionable pharmacological, dietary, and lifestyle-based strategies. As summarized in Fig. 5, these approaches can be conceptually divided into two stage-specific objectives: reinforcement of cytoprotective mechanisms in individuals who remain below the senescence threshold (“enhancing the shield”) and disruption of autophagy-dependent survival pathways in individuals who have crossed the threshold (“breaking the crutch,” senolysis).

**Table 1:** Clinical Trials targeting the autophagy-senescence axis (Trials 1–13 identified via primary search; Trials 14–15 included as supplementary context).

Trial ID	Intervention/Study Name	Status	Relevance to Threshold Model
NCT06186102	Spermidine (PolyCAD)	Active	**Shield:** Preventing cardiovascular senescence.
NCT04928963	Fasting Mimicking Diet	Recruiting	**Shield:** mTOR inhibition for immune rejuvenation.
NCT05421546	Spermidine (Immune)	Recruiting	**Shield:** Reversing T-cell immunosenescence.
NCT05343611	Choko-AGE (Diet + HIIT)	Recruiting	**Shield:** Cortisol reduction + exercise.
NCT07000708	Time-Restricted Eating	Recruiting	**Shield:** Metabolic boosting of vaccine response.
NCT03094546	Spermidine (SmartAge)	Completed	**Shield:** Polyamine supplementation for brain health.
NCT03754842	NAD + Precursors (NR + PT)	Completed	**Shield:** Sirtuin activation for stem cells.
NCT03326648	Protein + Strength Training	Completed	**Restoration:** Mitophagy/Lipophagy flux via exercise.
NCT02868021	Blood Flow Restriction	Completed	**Restoration:** Hypoxic stress triggers mitophagy.
NCT02577731	Trauma Assessment	Active	**Mechanism:** HSC failure due to autophagic stress.
NCT06313645	VICTORIA Study	Recruiting	**Biomarker:** Linking telomere damage to SASP.
NCT06320158	Sarcopenic Obesity	Recruiting	**Biomarker:** Autophagy defects in fat/muscle crosstalk.
NCT05555693	POCD Assessment	Recruiting	**Biomarker:** Brain metabolomics and autophagy.
NCT07144072^a^	UC-MSCs (HeXell-2020)	Recruiting	**Restoration:** Paracrine reversal of muscle senescence.
NCT02065245^a^	Allogeneic MSCs (CRATUS)	Completed	**Restoration:** Reducing frailty-associated inflammation.

Abbreviations: BFR, Blood Flow Restriction; CRATUS, Allogeneic Human Mesenchymal Stem Cells in Patients with Aging Frailty; FMD, Fasting Mimicking Diet; HeXell-2020, Umbilical Cord-Derived Mesenchymal Stem Cell Therapy; HIIT, High-Intensity Interval Training; HSC, Hematopoietic Stem Cell; MSC, Mesenchymal Stem Cell; mTOR, Mechanistic Target of Rapamycin; NAD+, Nicotinamide Adenine Dinucleotide; NCT, National Clinical Trial; NR, Nicotinamide Riboside; POCD, Post-Operative Cognitive Dysfunction; PolyCAD, Polyamine Treatment in Elderly Patients with Coronary Artery Disease; PT, Pterostilbene; SASP, Senescence-Associated Secretory Phenotype; UC-MSC, Umbilical Cord-Derived Mesenchymal Stem Cell.

a Supplementary trials included for context.

To provide a comprehensive translational framework, this portfolio also incorporates relevant evidence from two stem cell–based regenerative studies (**NCT07144072** and **NCT02065245**), which complement autophagy-centered interventions by addressing tissue repair and cellular replenishment. Collectively, current clinical efforts fall into three principal categories: (i) enhancement of cytoprotective autophagy for disease prevention, (ii) senolytic strategies targeting autophagy-dependent survival of senescent cells, and (iii) development and validation of biomarkers to stratify patients by disease stage and therapeutic responsiveness.

### 5.1 Enhancing the shield: prevention through autophagy induction

Interventions applied during the pre-threshold phase aim to restore or augment basal autophagic flux, thereby preventing cumulative cellular damage and delaying senescence onset.

#### 5.1.1 Metabolic reprogramming: resetting the nutrient sensors

The most evolutionarily conserved mechanism for autophagy induction is nutrient deprivation, which engages a coordinated nutrient-sensing switch. Reduced nutrient availability suppresses mTORC1 activity—an inhibitor of autophagy—while activating the energy sensor AMPK, thereby promoting autophagosome formation and lysosomal degradation.

##### 5.1.1.1 Fasting mimicking diet (FMD)

The phase I/II trial **NCT04928963** evaluates intermittent fasting-mimicking diet (FMD) cycles in pre-frail older adults. This strategy delivers transient starvation signals without the risks associated with chronic caloric restriction. Periodic reductions in insulin/IGF-1 signaling and mTORC1 activity are expected to stimulate autophagic clearance mechanisms. The proposed outcome is selective apoptosis of senescent, myeloid-biased white blood cells, followed by regenerative expansion of naïve hematopoietic stem cells upon re-feeding, thereby rejuvenating immune competence [65].

##### 5.1.1.2 Time-restricted eating (TRE)

The VITAL study (**NCT07000708**) investigates a less intensive metabolic intervention based on time-restricted eating. By confining food intake to a defined daily window, this approach induces cyclical AMPK activation during fasting periods, enhancing basal autophagy without prolonged nutrient deprivation. TRE is hypothesized to mitigate immunosenescence by eliminating functionally exhausted immune cells and improving vaccine responsiveness, as assessed by enhanced antibody production following influenza vaccination [66].

#### 5.1.2 Nutraceutical induction: pharmacological mimetics

For older individuals in whom fasting interventions may be contraindicated due to frailty or sarcopenia, nutraceuticals provide an alternative strategy. These agents function as caloric restriction mimetics, chemically targeting the same nutrient-sensing pathways—principally mTORC1 inhibition and AMPK activation—to induce autophagy while preserving nutritional intake.

##### 5.1.2.1 Polyamine supplementation: spermidine

Spermidine induces autophagy primarily through inhibition of acetyltransferases, resulting in deacetylation and activation of core autophagy execution proteins. This epigenetic and post-translational regulation establishes a sustained autophagic state without overt nutrient deprivation. Two ongoing clinical trials exemplify its translational relevance. The PolyCAD trial (**NCT06186102**) targets cardiovascular aging, testing the hypothesis that spermidine-induced autophagy promotes removal of dysfunctional mitochondria in cardiomyocytes, thereby improving myocardial remodeling. In parallel, the SmartAge study (**NCT03094546**) investigates whether enhancement of neuronal proteostasis via spermidine supplementation can facilitate clearance of neurotoxic protein aggregates in individuals with subjective cognitive decline [67].

##### 5.1.2.2 NAD + restoration

Age-associated decline in nicotinamide adenine dinucleotide (NAD^+^) compromises sirtuin activity, particularly SIRT1, an NAD^+^-dependent deacetylase that directly regulates autophagy through targets such as ATG5 and ATG7. The **NCT03754842** trial evaluates supplementation with nicotinamide riboside (NR) as a strategy to restore intracellular NAD^+^ levels and reactivate SIRT1 signaling. The anticipated outcome is enhancement of autophagy-dependent regenerative capacity in skeletal muscle satellite cells, thereby improving tissue repair in aging individuals [68].

#### 5.1.3 Lifestyle & exercise: mechanical and hypoxic stress as autophagy inducers

Physical stress represents a potent, non-pharmacological stimulus for autophagy, mediated through calcium signaling, transient ATP depletion, and activation of AMPK.

##### 5.1.3.1 Synergistic interventions

The Choko-AGE trial (**NCT05343611**) evaluates a dual-modality intervention combining polyphenol-rich chocolate supplementation with high-intensity interval training (HIIT). Acute ATP depletion during HIIT activates AMPK, thereby inducing autophagy. Concurrently, cocoa-derived polyphenols—particularly epicatechin—attenuate cortisol-mediated stress responses, which may otherwise suppress tissue repair. This synergistic strategy is designed to enhance exercise-induced autophagy while preserving muscle mass in aging populations.

##### 5.1.3.2 Hypoxic stress

The **NCT02868021** trial investigates blood flow restriction (BFR) training as an alternative autophagy-inducing stimulus. BFR creates localized hypoxia during low-intensity exercise, stabilizing hypoxia-inducible factor 1α (HIF-1α), which transcriptionally upregulates BNIP3, a key mitophagy receptor. This approach promotes selective elimination of damaged mitochondria with minimal mechanical load, making it particularly suitable for older individuals with limited exercise tolerance or osteoarthritis [69].

### 5.2 Breaking the crutch: stem cell–based modulation in the post-threshold phase

In the post-threshold state, persistent SASP signaling disrupts tissue homeostasis. Therapeutic strategies therefore shift from prevention toward active restoration and immunomodulation, with an emphasis on paracrine mechanisms rather than direct cell replacement.

#### 5.2.1 Paracrine rescue: secretome

The HeXell-2020 study (**NCT07144072**) exemplifies this paradigm shift by employing umbilical cord–derived mesenchymal stem cells (UC-MSCs). These cells exhibit high basal autophagic activity and function primarily as sources of regenerative paracrine signals. UC-MSC–derived exosomes and microvesicles deliver functional mRNAs, microRNAs, and proteins to aged host tissues, where they reactivate autophagy and suppress NF-κB–driven inflammatory signaling. This paracrine modulation effectively reverses the autophagy blockade that sustains senescence in post-threshold tissues [70].

#### 5.2.2 Anti-inflammatory immunomodulation

The CRATUS trial (**NCT02065245**) provided clinical validation of this approach, demonstrating that allogeneic MSC administration significantly reduces circulating TNF-α levels in frail older adults. By disrupting a central inflammatory node of the SASP, this intervention shifts tissues from a state of chronic low-grade inflammation (inflammaging) toward a regenerative microenvironment [71].

#### 5.2.3 Trauma as a stress-test of autophagic capacity

The **NCT02577731** study highlights limitations of regenerative strategies under extreme stress conditions. Acute trauma induces rapid proliferation of hematopoietic stem cells (HSCs), generating oxidative stress that must be resolved through autophagy. In aged individuals, insufficient autophagic capacity results in damage accumulation and premature HSC senescence rather than regeneration. Defining this failure threshold is essential for developing adjunctive therapies that preserve stem cell function during medical emergencies.

### 5.3 The diagnostic gap: validating biomarkers of autophagy-senescence crosstalk

Effective clinical deployment of the threshold model requires precise patient stratification. While autophagy induction is beneficial before the threshold is reached, it may exacerbate pathology once senescence is established. Robust biomarkers are therefore essential to guide intervention timing.

#### 5.3.1 The telomere-mitochondria axis

The VICTORIA study (**NCT06313645**) explores the mechanistic link between telomere attrition and mitochondrial dysfunction, focusing on telomeric repeat–containing RNA (TERRA). Altered TERRA expression following telomere shortening influences mitochondrial metabolism and inflammatory signaling. This trial aims to validate circulating TERRA as a biomarker of post-threshold senescence, particularly in atherosclerosis, where SASP-driven inflammation accelerates plaque vulnerability [72].

#### 5.3.2 Measuring flux versus static burden

A major limitation of current trials is reliance on static biomarkers that fail to capture dynamic autophagic activity. The Amazentis study (**NCT02472340**) addresses this gap by validating methodologies to quantify mitophagy flux in human skeletal muscle. This dynamic measurement distinguishes adaptive mitochondrial expansion from pathological accumulation caused by impaired clearance, enabling more accurate identification of patients likely to benefit from autophagy-modulating therapies [73].

### 5.4 Regulatory considerations and the drug-repurposing strategy

Despite a strong biological rationale, development of de novo anti-aging therapeutics faces regulatory barriers, as aging and senescence are not currently recognized as treatable indications by the FDA. Consequently, the field has increasingly adopted a drug-repurposing strategy, leveraging FDA-approved agents with established safety profiles to target the autophagy–senescence axis (Table 2).

**Table 2:** FDA-approved drugs that could be used in the autophagy-senescence paradigm.

Drugs	Brand/Manufacturer/Date)	Original FDA indications	Relevance to the threshold model	Ref.
Rapamycin (Sirolimus)	Rapamune/Wyeth (Pfizer)/Sept 15, 1999	Prophylaxis of kidney transplant rejection; lymphangioleiomyomatosis	**The “shield” (inducer):** The most potent pharmacological inducer of autophagy. Used to maintain the “pre-threshold” shield by inhibiting mTORC1.	[74]
Metformin	Glucophage/Bristol-Myers Squibb/Dec 29, 1994	Management of Type 2 DM.	**The “shield” (inducer):** Activates AMPK to simulate caloric restriction, preventing the accumulation of metabolic stress that triggers senescence.	[75]
Hydroxy chloroquine	Plaquenil/Sanofi-Synthelabo/Apr 18, 1955	Malaria, lupus erythematosus, and rheumatoid arthritis.	**The “starvation strategy” (inhibitor):** Blocks the “metabolic crutch” (autophagy) in post-threshold cells (e.g., cancer) to induce starvation.	[76]
Dasatinib	Sprycel/Bristol-Myers Squibb/June 28, 2006	CML, ALL.	**The “crutch breaker” (senolytic):** Combined with quercetin to selectively eliminate senescent cells by disabling their anti-apoptotic defense (SCAPs).	[77]
Trametinib	Mekinist/GlaxoSmithKline/May 29, 2013	Unresectable or metastatic melanoma (BRAF^V600 E/K^).	**The “SASP dampener":** Inhibits the MEK/ERK pathway, uncoupling the cell cycle arrest from the inflammatory secretory phenotype.	[78]

DM: Diabetes mellitus; CML: Chronic myeloid leukemia; ALL: Acute lymphoblastic leukemia.

This approach aligns directly with the biphasic structure of the threshold model. Agents such as rapamycin and metformin—originally approved for immunosuppression and diabetes, respectively—are repurposed to reinforce pre-threshold cytoprotective autophagy via modulation of the mTOR–AMPK axis. Conversely, post-threshold interventions focus on disrupting autophagy-dependent survival pathways. Hydroxychloroquine, an antimalarial agent, is used to inhibit lysosomal acidification in therapy-resistant cancers, while dasatinib is being evaluated for its senolytic potential. Together, these strategies enable rapid clinical translation of the threshold model, transforming established pharmacotherapies into precision geroprotective interventions.

### 5.5 Epigenetic fixation of autophagy reprogramming

Signaling mechanisms (e.g., mTOR/AMPK) initiate the transition from autophagy to senescence, but epigenetic modifications are required to maintain the post-threshold state [79]. Senescent cells display enduring DNA methylation and histone modification patterns that confine the autophagy network to a “secretory” state. This transition is often cemented by the promoter hypermethylation of key autophagy genes (e.g., *ULK1 and ATG5*), which prevents the restoration of efficient degradative flux even after the initial stress has been resolved [[80], [81], [82]]. This epigenetic “locking” ensures that the senescent phenotype persists and forces cells to rely on the aberrant, high-energy TASCC pathway for survival [23].

These findings elucidate a molecular foundation for novel treatment approaches that integrate senolytics with epigenetic modulators (e.g., HDAC or DNMT inhibitors) to “unlock” these states and reestablish therapeutic plasticity.

### 5.6 Heterogeneity: the challenge of distinct senotypes

Finally, it is crucial to recognize that senescence is not a singular cellular condition but rather a spectrum of many “senotypes” that vary based on the tissue of origin and the specific stressor that produced it (such as oncogene-driven, replicative, or therapy-induced senescence). Metabolic demands and reliance on specific autophagic mechanisms likely differ among distinct subpopulations. Determining which forms of autophagy (macroautophagy, CMA, or secretory autophagy) are predominant in each unique senotype remains crucial. Recognizing this heterogeneity is crucial for improving the threshold model; therapeutic interventions should be tailored to the specific pathological driver rather than employing a universal blockade, thus ensuring the targeting of pathogenic senescent cells while maintaining beneficial, stress-adaptive responses [79].

### 5.7 Sexual dimorphism: does the threshold differ by sex?

Current conceptualizations often treat the autophagy-senescence threshold as a universal standard, yet emerging evidence reveals profound sexual dimorphism. Sex hormones, particularly 17β-estradiol, are potent modulators of the autophagic machinery, signaling through AMPK to enhance mitochondrial quality control [83]. Consequently, pre-menopausal females likely possess a higher “structural threshold” for senescence compared to age-matched males, supported by superior mitophagy efficiency and redox regulation. However, this protection is not permanent: the abrupt loss of estrogen during menopause precipitates a steep, rapid collapse in autophagic flux, contrasting with the more gradual decline observed in males [84]. This bioenergetic divergence likely underpins the distinct trajectories of neurodegenerative and metabolic diseases between sexes [85]. Therefore, precision gerontology must abandon “one-size-fits-all” approaches and integrate sex as a biological variable to accurately define the distinct therapeutic windows for autophagy modulation.

## 6 Conclusion and future perspectives: navigating the senescence threshold

The relationship between autophagy and cellular senescence is not a binary opposition between cellular survival and death, but a dynamic, stage-dependent continuum shaped by stress intensity and disease progression. This review highlights autophagy as a context-dependent regulator with dual functionality: under physiological conditions, it preserves tissue integrity by suppressing senescence initiation, whereas beyond a critical damage threshold, it is repurposed to sustain the metabolic and inflammatory demands of established senescent cells. The threshold model proposed here provides a unifying framework to reconcile long-standing inconsistencies in the field and to rationalize apparently conflicting therapeutic observations.

Within this framework, autophagy activation emerges as a viable geroprotective strategy in the pre-threshold phase, as exemplified by interventions such as spermidine, rapamycin, and lifestyle-based metabolic reprogramming. Conversely, in post-threshold states—including therapy-resistant cancers and fibrotic or inflammatory pathologies—autophagy inhibition becomes necessary to disrupt senescent cell survival. Mechanistic insights into TASCC and organelle-specific dysfunctions, such as p53-mediated mitophagy suppression and aberrant nucleophagy activation, underscore that senescent cells are not metabolically inert but actively rewire intracellular signaling networks to evade elimination.

Successful clinical translation of these concepts requires resolution of the current diagnostic gap. As highlighted by emerging trials, including VICTORIA and Amazentis, progress toward dynamic and functional biomarkers is underway; however, indiscriminate modulation of autophagy remains potentially harmful. In particular, autophagy induction in individuals who have already crossed the senescence threshold risks amplifying the senescence-associated secretory phenotype, thereby accelerating tissue degeneration rather than restoring homeostasis.

The future of this field therefore lies in precision gerontology, the integration of mechanistic biology, stage-specific intervention, and biomarker-guided patient stratification. Development and validation of sensitive, minimally invasive indicators, such as circulating lncRNA TERRA, growth differentiation factor 15 (GDF15), and imaging-based assessments of mitophagy flux, will be essential for defining an individual's position along the senescence trajectory. Only through such stratification can the autophagy–senescence axis be safely and effectively targeted—employing restoration strategies before the threshold and elimination strategies after it. The era of treating aging as a monolithic process is ending; the next phase will focus on precise manipulation of the molecular decision circuitry that governs cellular fate.

## Funding

This study was supported by grants from the Basic Science Research Program through the 10.13039/501100003725National Research Foundation of Korea (RS-2023-00219399, RS-2023-00238051).

## CRediT authorship contribution statement

**Md Entaz Bahar:** Conceptualization, Data curation, Methodology, Writing – original draft. **Jin Seok Hwang:** Data curation, Writing – review & editing. **Trang Huyen Lai:** Methodology, Writing – review & editing. **Kazi-Marjahan Akter:** Formal analysis, Writing – review & editing. **Rizi Firman Maulidi:** Methodology, Writing – review & editing. **Deok Ryong Kim:** Conceptualization, Formal analysis, Funding acquisition, Supervision, Writing – original draft.

## Declaration of competing interest

The authors declare that there is no conflict of interest.

## Data Availability

No data was used for the research described in the article.
